# Clinical outcomes in recently diagnosed atrial fibrillation related to mitral stenosis compared to non-valvular atrial fibrillation

**DOI:** 10.3389/fcvm.2025.1555557

**Published:** 2025-06-18

**Authors:** Mohamed Aboelhassan, Hosam Hasan-Ali, Mohammed Taha Mohammed, Amr Ashry, Mohamed Aboel-Kassem F. Abdelmegid

**Affiliations:** ^1^ Department of Cardiology, Faculty of Medicine, Assiut University, Assiut, Egypt; ^2^ Department of Cardiothoracic Surgery, Faculty of Medicine, Assiut University, Assiut, Egypt

**Keywords:** mitral stenosis, atrial fibrillation, non-valvular atrial fibrillation, NVAF, rheumatic heart disease

## Abstract

**Background:**

Atrial fibrillation (AF) is the most common sustained arrhythmia in adults and is associated with significant morbidity and mortality. Mitral stenosis (MS) is common in developing countries, affecting the younger population and posing a risk of atrial fibrillation.

**Objectives:**

This study aims to delineate the clinical characteristics and poor outcomes in patients who have recent non-valvular AF (NVAF) compared with AF related to MS (MS-AF). Furthermore, it seeks to assess the healthcare resource utilization associated with the management of AF patients in the two groups.

**Patients and methods:**

This is a prospective observational cohort conducted on 84 patients with recent AF. The patients were divided into two groups: the NVAF group (patients with no prosthetic valves or moderate/severe MS) and the MS-AF group (patients with AF in the presence of moderate/severe MS). The clinical characteristics, stroke risk, and anticoagulation regimens were assessed. AF-related outcomes (strokes, hospitalizations, major bleeding, and mortalities) were monitored and compared between the two groups.

**Results:**

The mean age of the studied AF patients was 49.80 ± 16.31 years, ranging from 25 to 89 years. Patients with MS-AF were significantly younger than patients with NVAF. Hypertension was the most prevalent risk factor associated with AF. Smoking, heart failure, and hypertension were more prevalent among patients with NVAF. The NVAF group received less anticoagulants than the MS-AF group. There were no statistically significant differences between the two groups regarding the overall incidence of death, stroke, myocardial infarction, TIA, or hospital admissions. In the overall studied group, all-cause mortality was higher among AF patients with a history of heart failure or stroke.

**Conclusion:**

Patients with NVAF had a significantly greater incidence of cardiovascular disease risk factors. However, AF related to mitral stenosis was associated with comparable worse outcomes.

## Introduction

Atrial fibrillation (AF) is the most common sustained arrhythmia, and its incidence is increasing globally. It is associated with a substantial risk of stroke, heart failure, recurrent hospitalization, and death. It is also an important cause of healthcare burden and costs.

AF is broadly classified as either valvular or non-valvular, according to the recent ESC guidelines. The distinction between non-valvular atrial fibrillation (NVAF) and valvular atrial fibrillation—defined as AF in the presence of moderate-to-severe mitral stenosis (MS) or mechanical prosthetic heart valves—is clinically significant, as these groups differ not only in thromboembolic risk but also in recommended anticoagulation strategies (1). Globally, rheumatic heart disease remains the leading cause of mitral stenosis, with a particularly high prevalence in developing countries. Unlike degenerative mitral valve disease, rheumatic mitral stenosis tends to affect a younger demographic, often presenting in early adulthood. Patients with rheumatic mitral stenosis are also at a significantly increased risk of developing atrial fibrillation compared with individuals without structural mitral valve disease. Despite this elevated risk, they have been systematically excluded from most major clinical trials evaluating anticoagulation strategies in atrial fibrillation, leading to a gap in evidence-based management for this population.

The current study aims to evaluate the demographic data, worse outcomes, and healthcare resource utilization in the newly diagnosed AF related to mitral stenosis, compared with non-valvular AF.

## Methods

### Study population

This prospective observational cohort was conducted at a tertiary center (Assiut University Heart Hospital, Egypt) from July 2022 to July 2023. It enrolled patients presenting with recent-onset atrial fibrillation (3 months) in the presence of moderate-to-severe mitral stenosis (MS), along with a matched cohort of patients with recent NVAF patients.

All patients provided informed consent. The study was conducted in compliance with the principles of the Helsinki Declaration on human research and received approval from local institutional committees.

**Inclusion criteria:** all adult cases, male or female, aged 18 years or older at the time of enrollment, who presented with recent-onset AF.

**Exclusion criteria:** (1) AF occurring in specific clinical contexts, including secondary AF related to thyrotoxicosis, the postoperative period, or acute myocardial infarction, (2) patients with mechanical heart valves or valve disease (other than mitral valve) scheduled for surgical valve replacement, (3) coexisting medical conditions requiring long-term anticoagulation for reasons other than AF, or (4) current pregnancy or breastfeeding.

### Definitions

AF was defined according to the 2020 ESC Guidelines for the diagnosis and management of atrial fibrillation (1). NVAF was defined as AF in the absence of moderate/severe MS and prosthetic valves. MS severity was assessed according to the 2021 ESC/EACTS guidelines for the management of valvular heart disease (2). Moderate mitral stenosis is defined as MVA ≤2 cm^2^ and severe (or significant) MS MVA ≤1.5 cm^2^ as assessed by transthoracic echocardiography. Patients were classified into two groups: those with non-valvular atrial fibrillation (NVAF) and those with atrial fibrillation in the presence of moderate-to-severe mitral stenosis (MS-AF).

### Study outcomes and follow-up

Demographic, clinical, laboratory, medication, and echocardiographic parameters were compared between the two groups. All enrolled patients were followed up at 6 months and 1 year. Adverse outcomes were recorded and compared between the two groups. They included all-cause mortality, ischemic cerebrovascular stroke, transient ischemic attack (TIA), myocardial infarction, major and minor bleeding events, and hospitalization. Major bleeding was defined as any bleeding event associated with one or more of the following: a drop in hemoglobin of ≥2 g/dl within 24 h, the need for ≥2 units of packed red blood cells, bleeding in critical sites (intracranial, intraspinal, intraocular, intra-articular, pericardial, retroperitoneal, intramuscular with compartment syndrome), or death. Minor bleeding was defined as any bleeding that did not meet the criteria for major bleeding. Hospitalization was defined as any initial hospital admission (even if <24 h) related to atrial fibrillation or associated adverse outcomes. Medication prescription and management decisions, including rate and rhythm control strategies, were made in accordance with standard clinical practice. The economic burden of AF management was estimated by calculating total healthcare resource utilization costs, derived by multiplying each unit of service by its corresponding estimated cost. Healthcare utilization was assessed based on the total number of hospital admissions, length of hospital stays, outpatient visits, and the number of ECG and echocardiographic examinations performed.

Certain considerations have been addressed regarding the handling of missing data and participant dropouts. Patients with missing critical outcome data during the 12-month follow-up were excluded from the respective analyses. The overall rate of missing data was low (<5%) and evenly distributed between the MS-AF and NVAF groups. Therefore, no imputation methods were applied. Patients lost to follow-up were not included in survival or event-based outcome analyses.

### Statistical analysis

All statistical calculations were done by using Statistical Package for the Social Sciences (SPSS Inc., Chicago, IL, USA) version 22. Quantitative data were described using mean ± standard deviation (SD) and median with range, as appropriate. Qualitative data were summarized using frequencies (number of cases) and relative frequencies (percentages) when appropriate. Comparison of quantitative variables was done using Student’s *t*-test for normally distributed data and Mann–Whitney *U* test for non-normally distributed data. Friedman test was used for comparing continuous data over time. For comparing categorical data, Chi-square (*χ*^2^) test was performed. Fisher’s exact test was used when the expected frequency was <5. Kaplan–Meier's method with log-rank test was used for overall survival (OS) analysis. Hazard ratio (HR) with a 95% confidence interval (CI) and Cox regression analysis were calculated for the prediction of all causes of mortality risk factors among the studied participants. A *p*-value of <0.05 was considered statistically significant.

## Results

The study included 84 patients with recent-onset atrial fibrillation (within the past 3 months). This included 42 patients with AF and moderate/severe mitral stenosis (MS-AF group) and a matched number of non-valvular AF patients (NVAF group). The mean age of all AF cases was 49.80 ± 16.31 (ranging from 25 to 89 years old). Hypertension was the most prevalent risk factor associated with AF (28 cases, 33.3%). Patients with MS-AF were significantly younger than patients with NVAF. History of smoking, heart failure, or hypertension was more prevalent among patients with NVAF (*p* = 0.046, 0.010, and <0.001, respectively). The two groups were comparable in terms of body mass index (BMI), gender, and the remaining risk factors associated with AF (*p* > 0.05 for all). Patients' characteristics in both groups are shown in Table 1.

**Table 1:** Patients’ characteristics in the studied groups.

Patients’ characteristics	Total (*n* = 84)		MS-AF (*n* = 42)		Non-valvular AF (*n* = 42)		*p-*value
Age (years)	49.80 ± 16.31		41.98 ± 13.76		57.62 ± 14.97		**<0**.**001**
Age groups, *n* (%)							**<0**.**001**
18–40	36	(42.9)	27	(64.3)	9	(21.4)	
41–59	23	(27.4)	11	(26.2)	12	(28.6)	
60–74	16	(19.0)	3	(7.1)	13	(31.0)	
≥75	9	(10.7)	1	(2.4)	8	(19.0)	
Sex, *n* (%)							0.081
Male	42	(50.0)	17	(40.5)	25	(59.5)	
BMI (kg/m^2^)	21.52 ± 3.91		21.90 ± 3.60		21.14 ± 4.21		0.375
Risk factors, *n* (%)							
Family history AF	3	(3.6)	1	(2.4)	2	(4.8)	1
Smoking	15	(17.9)	4	(9.5)	11	(26.2)	**0**.**046**
Heart failure	15	(17.9)	3	(7.1)	12	(28.6)	**0**.**010**
Hypertension	28	(33.3)	6	(14.3)	22	(52.4)	**<0**.**001**
Previous stroke	7	(8.3)	4	(9.5)	3	(7.1)	1
Diabetes mellitus	21	(25.0)	11	(26.2)	10	`(23.8)	0.801
CKD	9	(10.7)	2	(4.8)	7	(16.7)	0.156
CAD	15	(17.9)	7	(16.7)	8	(19.0)	0.776
COPD	9	(10.7)	3	(7.1)	6	(14.3)	0.483
PAD	2	(2.4)	1	(2.4)	1	(2.4)	1
Dyslipidemia	8	(9.5)	2	(4.8)	6	(14.3)	0.265
Thyroid disease	1	(1.2)	0	(0.0)	1	(2.4)	1

Bold values indicate significant *p* value.

AF, atrial fibrillation; BMI, body mass index; CKD, chronic kidney disease; CAD, coronary artery disease; COPD, chronic obstructive pulmonary disease; MS-AF, AF related to mitral stenosis; PAD, peripheral arterial disease. Quantitative data are presented as mean ± SD, and qualitative data are presented as number (percentage). Significance defined by *p* < 0.05.

Patients with MS-AF had significantly larger left atrial volume than patients with NVAF (48.1 ± 4.9 cm^3^ vs. 41.3 ± 5.2 cm^3^, respectively; *p* < 0.001), while left ventricle ejection fraction (LVEF) (%) showed no significant difference between the two groups (*p* = 0.059). Systolic blood pressure was significantly higher in patients with NVAF. Stroke risk in patients with NVAF, as assessed by CHA_2_DS_2_-VASc score, showed that most of the patients (64.3%) were at high risk (Table 2).

**Table 2:** Clinical and echocardiographic characteristics in studied groups.

Variables	MS-AF (*n* = 42)	Non-valvular AF (*n* = 42)		*p-*value
Echocardiographic characteristics				
LVEF (%)	58.57 ± 5.99	53.95 ± 14.44		0.059
LA volume (cm^3^)	48.1 ± 4.9	41.3 ± 5.2		**<0**.**001**
Blood pressure and HR, median (range)				
SBP (mmHg)	110 (90–150)	130 (90–180)		**0**.**010**
DBP (mmHg)	80 (60–90)	80 (50–110)		0.343
HR (beats/min)	100 (80–150)	100 (45–140)		0.372
CHA_2_DS_2_-VASc score				
Low risk (0)	NA	4	(9.5%)	
Moderate risk (1)	NA	11	(26.2%)	
High risk (≥2)	NA	27	(64.3%)	

Bold values indicate significant *p* value.

CHA_2_DS_2_-VASc = congestive heart failure, hypertension, age ≥75 years, diabetes mellitus, stroke, vascular disease, age 65–74 years, sex category (female). HR, heart rate; LVEF, left ventricle ejection fraction; MS-AF, AF related to mitral stenosis; NA, not applicable. Quantitative data are presented as mean ± SD or median (range), and qualitative data are presented as number (percentage). Significance defined by *p* < 0.05.

Oral anticoagulants were used in all MS-AF patients and 27 (64.3%) NVAF patients (*p* < 0.001). Warfarin was used in all MS-AF patients and in 19 NVAF patients (70.4% of those who received anticoagulation). Novel oral anticoagulants (NOACs) were used in none of the MS-AF patients and in eight NVAF patients (29.6% of those who received anticoagulants). Patients with MS-AF were more likely to receive digoxin (*p* = 0.027) and diuretics (*p* = 0.001), while patients with NVAF were more likely to receive angiotensin-converting enzyme (ACE) inhibitors (*p* = 0.001). Medications received by the studied groups are shown in Table 3.

**Table 3:** Medication received by the studied groups.

Medications	MS-AF AF (*n* = 42)		Non-valvular (*n* = 42)		*p-*value
Anticoagulant	42	(100.0)	27	(64.3)	**<0**.**001**
Type of anticoagulant					**<0**.**001**
Warfarin	42	(100.0)	19	(70.4)	
NOACs	0	(0.0)	8	(29.6)	
Aspirin	7	(16.7)	12	(28.6)	0.192
Beta-blocker	38	(90.5)	32	(76.2)	0.079
CCB	5	(11.9)	11	(26.2)	0.095
Digoxin	23	(54.8)	13	(31.0)	**0**.**027**
ACE inhibitor	2	(4.8)	18	(42.9)	**<0**.**001**
ARBs	1	(2.4)	3	(7.1)	0.616
ARNI	0	(0.0)	2	(4.8)	0.494
Diuretics	34	(81.0)	20	(47.6)	**0**.**001**
Other medication					0.054
Penicillin	4	(9.5)	0	(0.0)	
Amiodaron	1	(2.4)	0	(0.0)	
Carbimazol	0	(0.0)	1	(2.4)	
Chemotherapy	1	(2.4)	0	(0.0)	

Significance defined by *p* < 0.05. Bold values indicate significant *p* value.

ACE, angiotensin-converting enzyme; ARBs, angiotensin receptor blockers; ARNI, angiotensin receptor neprilysin inhibitor; CCB, calcium channel blocker; MS-AF, AF related to mitral stenosis; NOAC, novel oral anticoagulants.

In AF patients who received warfarin, there was no significant difference between the two groups regarding the therapeutic range (TTR) of anticoagulation (assessed by prothrombin time, prothrombin concentration, and INR) at baseline, 6 months, and 1 year**.** The percentage of days within the therapeutic range was 61% in MS-AF and 50% in NVAF patients, with no significant difference (*p* = 0.650) (Table 4).

**Table 4:** INR follow-up and time in therapeutic range (TTR) for patients who received warfarin.

INR level	MS-AF		Non-valvular AF		*p*-value
Baseline	25	(59.5%)	10	(52.6%)	0.920
Below therapeutic range					
Within therapeutic range	12	(29.3%)	7	(36.8%)	
Above therapeutic range	5	(12.2%)	2	(10.5%)	
After 6 months	14	(34.1%)	8	(42.1%)	0.652
Below therapeutic range	19	(46.3%)	9	(47.4%)	
Within therapeutic range					
Above therapeutic range	8	(19.5%)	2	(10.5%)	
After 12 months	7	(18.9%)	3	(21.4%)	1
Below therapeutic range	25	(67.6%)	10	(71.4%)	
Within therapeutic range					
Above therapeutic range	5	(13.5%)	1	(7.1%)	
Percent day within therapeutic range	61% (20–100)		50% (10–85)		0.650

The overall incidence of mortality, stroke, transient ischemic attacks, myocardial infarction, and hospital admissions was high among all AF patients, with a total of 11 deaths (eight cardiovascular, two non-cardiovascular, and one with unknown cause of death), seven strokes, and 47 hospitalizations during the follow-up period. There was no statistically significant difference between the two groups regarding deaths (11.9% vs. 14.3%, *p* = 0.746), stroke (7.1% vs. 9.5%, *p* = 1), transient ischemic attacks (0.0% vs. 7.1%, *p* = 0.241), myocardial infarction (4.8% vs. 2.4%, *p* = 1), or cardiac-related hospitalizations (61.9% vs. 50.0%, *p* = 0.272) for MS-AF and NVAF patients (Table 5). The incidence of major and minor bleeding was also comparable between the two groups, with no significant difference (*p* = 0.483).

**Table 5:** Comparison of outcomes between the studied groups.

One-year outcome	MS-AF (*n* = 42)		Non-valvular (*n* = 42)		*p-*value
All-cause mortality	5	(11.9)	6	(14.3)	0.746
Causes of death					0.697
Cardiovascular death	3	(60.0)	5	(83.3)	
Non-cardiovascular death	1	(20.0)	1	(16.7)	
Unknown cause	1	(20.0)	0	(0.0)	
Stroke	3	(7.1)	4	(9.5)	1
Myocardial infarction	2	(4.8)	1	(2.4)	1
Transient ischemic attack	0	(0.0)	3	(7.1)	0.241
Major bleeding	2	(4.8)	1	(2.4)	1
Minor bleeding	5	(11.9)	7	(16.7)	0.533
Hospitalization	26	(61.9)	21	(50.0)	0.272

Qualitative data are presented as number (percentage). Significance defined by *p* < 0.05.

**Figure 1:**
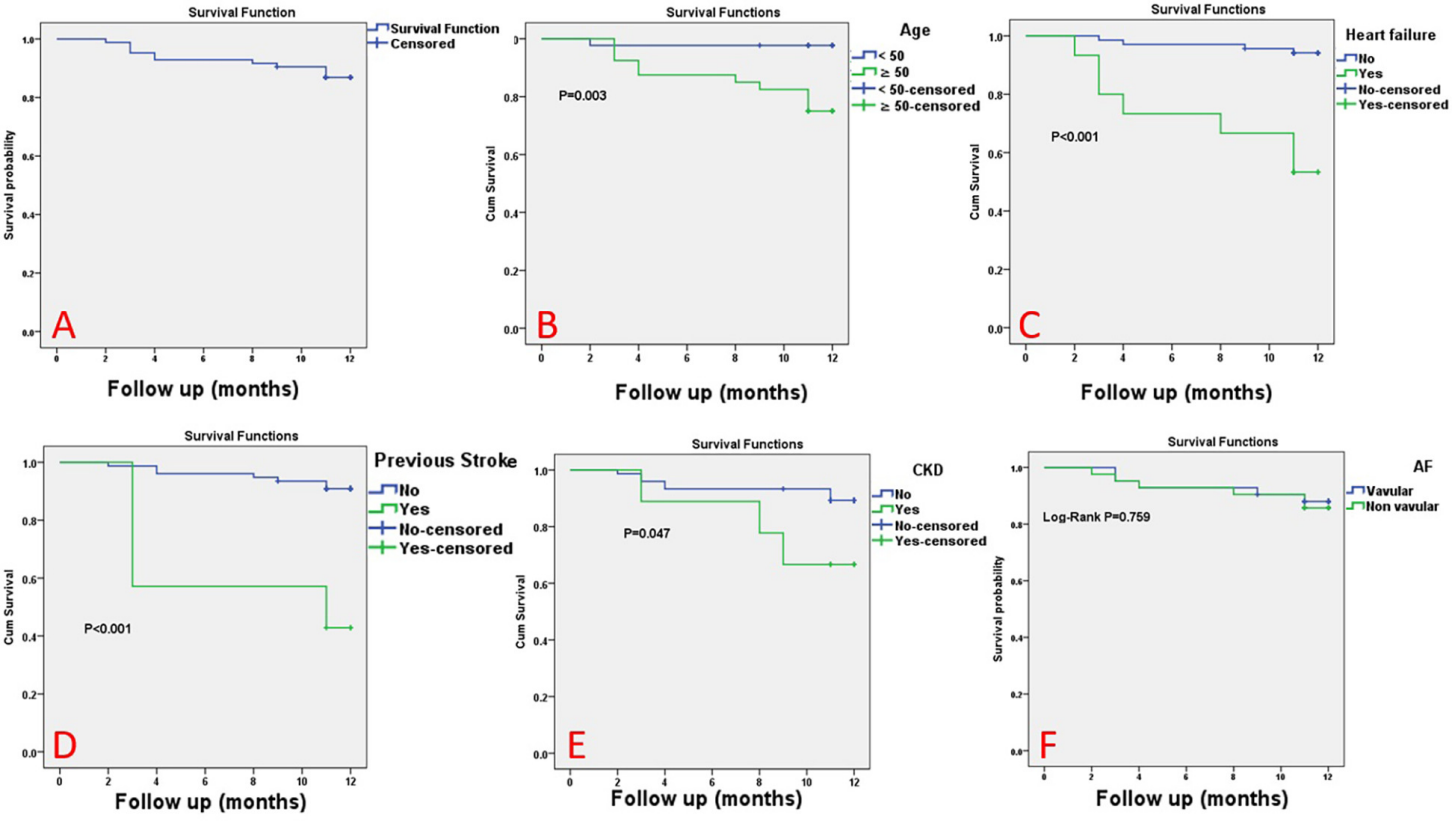
The Kaplan–Meier survival curve for the studied patients. The Kaplan–Meier survival curve for all studied atrial fibrillation (AF) patients **(A)**, according to age **(B)**, heart failure **(C)**, previous stroke **(D)**, chronic kidney disease **(E)**, and type of AF (non-valvular AF vs. MS-related AF) **(F)**.

Among the whole studied AF patients, the mortality rate was 13.1% at a mean follow-up of 11 months. According to Kaplan–Meier analysis, the overall survival (OS) rate at 11 months was 86.9%. Among all clinical parameters, patient age and history of heart failure, previous stroke, and/or chronic kidney disease (CKD) were observed to significantly affect the overall survival of the studied participants. However, the overall survival was not affected by the type of AF (MS-AF vs. NVAF). Survival analysis of variable parameters is shown in Table 6 and Figure 1.

**Table 6:** Overall survival analysis according to demographic and clinical parameters.

Demographic and clinical parameters	OS (12 months)	
	Estimate ± SE	*p-*value
Age (years)		**0**.**003**
<50	97.7 ± 2.2	
≥50	75.0 ± 6.8	
Sex		0.739
Male	88.1 ± 5.0	
Female	85.6 ± 5.4	
Smoking		0.424
No	85.4 ± 4.3	
Yes	93.3 ± 6.4	
Heart failure		**<0**.**001**
No	94.2 ± 2.8	
Yes	53.3 ± 12.9	
Hypertension		0.836
No	87.4 ± 4.4	
Yes	85.7 ± 6.6	
Previous stroke		**<0**.**001**
No	90.9 ± 3.3	
Yes	42.9 ± 18.7	
Diabetes mellitus		0.413
No	88.9 ± 4.0	
Yes	81.0 ± 8.6	
CKD		**0**.**047**
No	80.3 ± 3.6	
Yes	66.7 ± 15.7	
CAD		0.376
No	88.3 ± 3.9	
Yes	80.0 ± 10.3	
COPD		0.857
No	86.6 ± 3.9	
Yes	88.9 ± 10.5	
Dyslipidemia		0.267
No	88.1 ± 3.7	
Yes	75.0 ± 15.3	
Type of AF		0.759
MS-AF	88.0 ± 5.0	
NVAF	85.7 ± 5.4	

Significance defined by *p* < 0.05. Bold values indicate significant *p* value.

AF, atrial fibrillation; BMI, body mass index; CKD, chronic kidney disease; CAD, coronary artery disease; COPD, chronic obstructive pulmonary disease; MS-AF, AF related to mitral stenosis; NVAF, non-valvular AF.

According to multivariate analysis, AF patients with a history of heart failure and/or stroke had a greater likelihood of death. Among all AF patients, a history of heart failure was associated with a 10-fold increase in the risk of mortality (OR = 9.512, 95% CI: 12.169–41.706, *p* = 0.003), while a history of stroke was associated with a ninefold increase in the risk of mortality (OR = 8.773, 95% CI: 1.951–39.426, *p* = 0.005) (Table 7).

**Table 7:** Cox regression analysis for prediction of death among the studied patients.

Variables	Multivariate analysis		
	HR	95% CI	*p-*value
Age (years)			
<50	Ref		
≥50	3.397	0.363–31.753	0.284
Heart failure			
No	Ref		
Yes	9.512	2.169–41.706	**0**.**003**
History of stroke			
No	Ref		
Yes	8.773	1.951–39.426	**0**.**005**
CKD			
No	Ref		
Yes	0.978	0.230–4.165	0.976

Bold values indicate significant *p* value.

CI, confidence interval; HR, hazard ratio. A *p*-value of ≤0.05 is significant.

Among all studied patients, 55 patients (65.5%) required inpatient readmission (46 cases for once, and 9 cases for two times). An echocardiography exam was performed once for 68 patients (81%) and twice for 16 patients (19%). The median hospital stay was two days (range, 0–12 days), the median number of outpatient visits was three (range, 0–8 times), and the median number of ECGs was four (range, 1–8 times). No significant differences were observed between the two groups regarding overall healthcare resource utilization (*p* > 0.05, for all), as shown in Table 8.

**Table 8:** Healthcare resource utilization in both groups.

Outcome	Total (*n* = 84)		MS-AF (*n* = 42)		Non-valvular (*n* = 42)		*p-*value
Inpatient admission							0.214
0	29	(34.5)	11	(26.2)	18	(42.9)	
1	46	(54.8)	27	(64.3)	19	(45.2)	
2	9	(10.7)	4	(9.5)	5	(11.9)	
No. of echocardiography exams							0.578
1	68	(81.0)	33	(78.6)	35	(83.3)	
2	16	(19.0)	9	(21.4)	7	(16.7)	
Median hospital stays (days)	2 (0–12)		2 (0–9)		2 (0–12)		0.514
Median outpatient visits	3 (0–8)		4 (0–7)		3 (0–8)		0.455
ECG follow-up times	4 (1–8)		4 (2–8)		5 (1–6)		0.632

Quantitative data are presented as median (range).

## Discussion

This single-center study, conducted in Egypt, aimed to compare the epidemiological characteristics and clinical outcomes of atrial fibrillation (AF) related to moderate-to-severe mitral stenosis (MS-AF) with those of non-valvular atrial fibrillation (NVAF). A total of 84 newly diagnosed AF patients were enrolled, comprising 42 consecutive patients with MS-AF and a matched cohort of 42 patients with NVAF. The mean age of the study population was 49.8 years, with the MS-AF group being significantly younger than the NVAF group. The overall male-to-female ratio was 1:1; however, male predominance was observed in the NVAF group, while females predominated in the MS-AF group. A history of smoking, heart failure, and hypertension was more frequently observed among patients with NVAF. In alignment with prior studies (3–6), hypertension emerged as the most prevalent risk factor for AF in the overall cohort.

Rheumatic fever remains the leading global cause of mitral stenosis, with a declining prevalence in developed countries; however, it continues to represent a significant health burden in many developing regions (7–9). Advancing age is a well-established risk factor for AF, which is frequently associated with a variety of underlying etiologies and comorbid conditions (7–9). In contrast, rheumatic mitral stenosis typically affects younger individuals and is strongly associated with a high incidence of AF, primarily due to chronic left atrial pressure and volume overload (10). In the present study, patients with MS-AF demonstrated significantly greater left atrial volume compared with those with NVAF.

Although AF is generally more common in men—with European data indicating incidence rates roughly 20% higher in men than in women (7–11)—our cohort showed a predominance of mitral stenosis–related AF in women. This sex difference likely reflects the higher prevalence of rheumatic mitral stenosis among females (12). Notably, many of these women are of childbearing age, placing them at increased risk of maternal cardiovascular complications and posing distinctive challenges for stroke prevention and anticoagulation management during pregnancy.

At 1-year follow-up, the overall incidence rates of all-cause mortality, stroke, and major bleeding were notably high, occurring in 13.1%, 8.3%, and 3.6% of patients, respectively, with no statistically significant differences observed between the MS-AF and NVAF groups. In patients with NVAF, the risk of stroke and thromboembolism is well recognized to increase with age and higher CHA₂DS₂-VASc scores (13); in our study, 64.3% of NVAF patients had scores ≥2, indicating elevated thromboembolic risk. Despite being significantly younger, patients with MS-AF demonstrated comparable rates of stroke and mortality to those with NVAF. This finding is supported by data from the Framingham Heart Study, which reported that the presence of mitral stenosis in patients with AF increases the risk of stroke by over 20-fold (14). Similarly, a study from sub-Saharan Africa reported no significant differences in stroke or mortality between patients with valvular AF (including mitral stenosis and prosthetic valves) and those with NVAF (15).

High early mortality following AF diagnosis has also been documented in several large-scale observational cohorts. In a community-based study, mortality was highest within the first 4 months of diagnosis (16), and another registry reported that 30-day mortality following newly diagnosed AF exceeded rates observed in subsequent months (17). A population-based cohort of older adults with newly diagnosed AF showed a 1-year mortality rate of 19.5% (18). Furthermore, findings from a Kenyan study showed 12-month mortality and stroke rates of 10% and 5% in patients with valvular AF and 15% and 5% in those with NVAF, respectively (15). The elevated stroke incidence reported in the current study aligns with previously published data from several sub-Saharan African settings (3, 19–21).

Overall survival in the study cohort was significantly influenced by patient age and a history of heart failure, prior stroke, and/or chronic kidney disease (CKD). On multivariate analysis, only a history of heart failure and/or stroke remained independently associated with increased mortality risk. Stroke and systemic embolism have been reported to account for approximately 10% of all deaths among patients with atrial fibrillation (22–24). In the ROCKET AF trial, the Kaplan–Meier estimated mortality rates were 4.2% at 1 year and 8.9% at 2 years, with deaths more frequently occurring among older patients, males, and those with a history of heart failure, vascular disease, or impaired renal function (25). In the same analysis, independent predictors of increased mortality included reduced creatinine clearance, chronic obstructive pulmonary disease, male sex, peripheral vascular disease, advanced age, diabetes, heart failure, elevated heart rate, prior stroke or transient ischemic attack, and geographic location (Latin America) (25).

Oral anticoagulation therapy has been consistently shown to reduce the risk of stroke and improve survival in patients with AF who are at elevated thromboembolic risk (26–28). In patients with rheumatic mitral valve stenosis and/or mechanical prosthetic heart valves, vitamin K antagonists (VKAs) remain the only anticoagulant class with established safety and efficacy. In contrast, for patients with non-valvular atrial fibrillation, current guidelines recommend non-vitamin K antagonist oral anticoagulants (NOACs) as the preferred option over VKAs due to their favorable risk–benefit profile (1).

All patients with mitral stenosis and atrial fibrillation were treated with warfarin, whereas 70.4% of non-valvular atrial fibrillation patients who were anticoagulated also received warfarin. Among MS-AF patients on warfarin, the proportion of individuals within the therapeutic INR range remained suboptimal: 29.3% at baseline, 46.3% at 6 months, and 67.6% at the 12-month follow-up. The overall percentage of time spent within the therapeutic range (TTR) during follow-up was 61%, which is below the recommended threshold.

Although warfarin remains a well-established and effective oral anticoagulant, its utility is limited by a narrow therapeutic window, requiring frequent INR monitoring and individualized dose adjustments. Clinical evidence consistently demonstrates that maintaining a TTR above 70% is critical for maximizing efficacy and minimizing adverse events. Suboptimal TTR has been associated with increased risks of both thromboembolic complications and bleeding. Even modest improvements in TTR can lead to meaningful reductions in adverse outcomes and improve overall clinical care. Maintaining a high TTR is crucial in patients receiving warfarin, as suboptimal TTR is strongly associated with increased risks of thromboembolic and hemorrhagic events (28). Given the high prevalence of mitral stenosis–associated atrial fibrillation in our region, optimizing anticoagulation control is not only clinically important but also a pressing public health priority, warranting structured interventions to improve INR monitoring, patient adherence, and long-term outcomes.

Although the overall use of anticoagulation in our cohort was comparable to rates reported in global AF registries and observational studies (29–31), our real-world data highlight a pattern of underutilization of oral anticoagulants and preferential use of warfarin over NOACs among patients with non-valvular AF. This discrepancy likely reflects multifactorial barriers to optimal anticoagulation, including financial constraints, limited access to NOACs, concerns about adherence, and physician-related hesitancy in adopting guideline-directed therapies.

Despite the relatively small sample size, the elevated stroke and mortality rates observed may reflect a higher baseline risk of AF-related adverse outcomes in our population, potentially influenced by delays in diagnosis, suboptimal anticoagulation control, or systemic healthcare limitations. Moreover, previous studies have identified racial and ethnic disparities in AF outcomes, suggesting that sociodemographic and regional factors may contribute to worse prognoses in certain populations (32).

AF imposes a substantial economic burden on healthcare systems, primarily driven by hospitalizations, stroke-related complications, and reduced productivity (33–35). Globally, AF-related hospital admissions have shown a marked upward trend over recent years (36). This burden is particularly pronounced in low- and middle-income countries, where limited healthcare infrastructure and financial constraints amplify the social and economic consequences, especially among younger patients affected by AF. In the current study, hospitalization rates were notably high, reaching 61.9% in the MS-AF group and 50% in the NVAF group. However, overall healthcare resource utilization—including hospital readmissions, length of stay, outpatient clinic visits, and the frequency of ECG and echocardiographic assessments—did not differ significantly between the two groups.

The higher prevalence of rheumatic MS may contribute to the increased prevalence of AF seen in developing countries such as Egypt. AF associated with rheumatic MS predominantly affects a younger demographic, yet demonstrates adverse outcomes that are comparable to, if not worse than, those seen in non-valvular AF. The high rates of stroke and mortality observed in this study underscore the serious public health implications of AF in our population. These findings emphasize the urgent need for comprehensive, community-based strategies aimed at early detection, primary prevention, and consistent implementation of evidence-based guidelines for AF management. Enhancing public health awareness, improving access to diagnostic tools, and optimizing long-term care pathways may collectively help mitigate the burden of AF in resource-limited settings.

## Conclusion

Newly diagnosed atrial fibrillation is associated with high 1-year rates of stroke and mortality. Mitral stenosis–related AF, despite affecting a younger population with fewer cardiovascular risk factors and being managed with anticoagulation, demonstrates comparable adverse outcomes and healthcare resource utilization to non-valvular AF, highlighting the need for tailored management strategies in this subgroup.

## Data Availability

The raw data supporting the conclusions of this article will be made available by the authors, without undue reservation.
